# Glial contributions to visceral pain: implications for disease etiology and the female predominance of persistent pain

**DOI:** 10.1038/tp.2016.168

**Published:** 2016-09-13

**Authors:** K N Dodds, E A H Beckett, S F Evans, P M Grace, L R Watkins, M R Hutchinson

**Affiliations:** 1 Discipline of Physiology, School of Medicine, University of Adelaide, Adelaide, SA, Australia; 2 Discipline of Pharmacology, School of Medicine, University of Adelaide, Adelaide, SA, Australia; 3 Pelvic Pain SA, Norwood, SA, Australia; 4 Department of Psychology and Neuroscience, Center for Neuroscience, University of Colorado Boulder, Boulder, CO, USA; 5 ARC Centre of Excellence for Nanoscale BioPhotonics, University of Adelaide, Adelaide, SA, Australia

## Abstract

In the central nervous system, bidirectional signaling between glial cells and neurons (‘neuroimmune communication') facilitates the development of persistent pain. Spinal glia can contribute to heightened pain states by a prolonged release of neurokine signals that sensitize adjacent centrally projecting neurons. Although many persistent pain conditions are disproportionately common in females, whether specific neuroimmune mechanisms lead to this increased susceptibility remains unclear. This review summarizes the major known contributions of glia and neuroimmune interactions in pain, which has been determined principally in male rodents and in the context of somatic pain conditions. It is then postulated that studying neuroimmune interactions involved in pain attributed to visceral diseases common to females may offer a more suitable avenue for investigating unique mechanisms involved in female pain. Further, we discuss the potential for primed spinal glia and subsequent neurogenic inflammation as a contributing factor in the development of peripheral inflammation, therefore, representing a predisposing factor for females in developing a high percentage of such persistent pain conditions.

## From ‘hysteria' to a molecular understanding of female pain

Historical descriptions of chronic debilitating pain without obvious visible cause were originally restricted to females, and dated back over 2000 years to the era of renowned Greek physician Hippocrates (460–370 BC). Episodes of severe emotional and physical distress in women were diagnosed as ‘hysteria', a condition attributed to the movement of the uterus outside of the pelvis (the ‘wandering womb').^1^ Towards the end of the nineteenth century, the stigma surrounding female hysteria diminished owing to accumulating evidence that men could also suffer from persistent pain, work which was largely pioneered by Sigmund Freud (1856–1939).^2^ Considering pain as sex-independent in this context, along with general medical advances from the mid-twentieth century, has contributed to an immense expansion in our understanding of the mechanisms underlying the development of persistent pain. Notably, this is now known to involve bidirectional signaling between neurons and glia within the central nervous system (CNS).

However, a key discrepancy that remains in the literature is the clear over-representation of females among patients with persistent pain. There is an almost unanimous consensus that women are not only more sensitive in detecting painful stimuli, but are also the predominant sex with the most common painful disorders.^3, 4, 5, 6^ This includes, but is not limited to, conditions associated with neuropathic pain, musculoskeletal pain (such as back pain, fibromyalgia, osteoarthritis and complex regional pain syndrome), orofacial pain (including temporomandibular joint pain), abdominal and pelvic pain (such as irritable bowel syndrome, painful bladder syndrome and dyspareunia) and headache/migraine.^5^

Extensive epidemiological, clinical and experimental evidence implicates several biopsychosocial factors as contributing to the disparity in pain susceptibility across the sexes.^4^ Despite this, a dichotomy exists in the pain research field at large, where the vast majority of preclinical studies have characterized pain models using male subjects only.^7^ Moreover, evidence implicating neuroimmune signaling in the development of persistent pain has primarily been acquired using animal models of neuropathic and somatic inflammatory pain. This has included, but is not restricted to, muscle inflammation, spinal cord injury, peripheral nerve injury, arthritis, bone cancer and chemotherapy. Although many of these pathologies are important for understanding female pain, there is a lack of research into the large number of female-dominant conditions that stem from the viscera. Consequently, the specific biological mechanisms underlying the predisposition of females to persistent pain remain elusive.

It is possible that past research generalizing nociceptive mechanisms across the sexes has limited our approach in effectively treating female pain. Is it appropriate to assume that females process pain via identical mechanisms to males? Can we learn from, adapt and update aspects of the ancient Greek philosophy, by regarding female pain as a fundamentally distinct entity? And, to what extent do the sex-specific anatomical and neuroendocrine systems influence the heightened sensitivity of females to persistent pain?

To consider these questions, this review provides a summary of neuroimmune contributions, specifically those provided by astrocytes and microglia, to persistent pain signaling within the spinal cord. The concept that female sex hormones may modulate central neuroimmune signaling is then discussed, and that variations in these processes may have relevance for female-dominant pain conditions, as exemplified by several visceral inflammatory diseases. In addition, the dorsal root reflex is re-explored as a central driver of peripheral neurogenic inflammation, leading to the hypothesis that sensitized spinal glia might contribute to, and predispose, a subpopulation of females to persistent inflammatory pain.

## Persistent pain arises from central sensitization

Pain is a complex, unpleasant sensory and emotional experience that arises in response to, or is described in terms of, tissue damage.^8^ Distinct from the well-established protective and adaptive functions of acute pain, pain persisting beyond tissue healing is maladaptive and serves no known physiological function. In contrast to acute pain, the mechanisms involved in the development and maintenance of persistent pain are not fully understood. One potential mechanism that has received detailed investigation is the process of ‘central sensitization', whereby long-lasting molecular changes cause amplification of pain signaling by nociceptive neurons within the CNS. Central sensitization can include conditions of both hyperalgesia (heightened pain to a previously noxious stimulus) and allodynia (pain caused by a normally innocuous stimulus).^9, 10^ It is now acknowledged that the development of central sensitization engages not only neuronal, but also glial processes. Hence, the following sections outline the rationale for considering persistent pain to be a ‘gliopathy',^11^ in addition to the previously described ‘neuropathy'.

## Glia and the tetrapartite synapse support the maintenance of CNS homeostasis

Glia are a non-neuronal, immune-like cell population that constitute the vast majority of cells within the CNS. They comprise satellite glial cells in the ganglia, and microglia, astrocytes and oligodendrocytes within the spinal cord and brain. The anatomical co-localization of astrocytes and microglia in the spinal cord, combined with pre- and postsynaptic neurons, forms a key site of interaction termed the ‘tetrapartite synapse'.^12, 13^ Each cell within this functional unit reciprocally signals to another, contributing to a ‘neuroimmune communication' that allows glia to respond rapidly to disruptions in neuronal signaling.^14, 15^ The reactivity state and control of astrocytes and microglia is therefore critical in maintaining healthy CNS activity.

## Dysregulation of healthy glial activity contributes to the development of persistent pain

Following injury and aberrant nociceptive events, microglia and astrocytes increase their expression and secretion of various proinflammatory cytokines and chemokines.^15^ The stimulation of glial cells can occur by neurokine products released as a result of tissue injury, or by neurotransmitters released from activated neurons. Many of the proinflammatory responses of glia are important in protecting against challenges that disrupt the homeostatic balance of the CNS, such as during the sickness response—a constellation of adaptive behaviors and physiological responses that promote recovery from illness.^16^ However, under certain conditions, glial reactivity is not advantageous and can instead be detrimental to neuronal function, such as during the manifestation of persistent pain.

In response to strong or persistent receptor stimulation, microglia switch from a surveillance state to an active response state, and astrocytes transition from a regulatory to reactive state.^11^ Under these circumstances, the release of proinflammatory mediators by glia can contribute to ongoing nociception, by inducing long-lasting plastic changes of synaptic connectivity that enhances the transmission of ascending nociceptive information. As such, glia and their products are sufficient to create exaggerated pain. This has been shown where intrathecal transfer of highly reactive microglia alone, or injection or induction of their proinflammatory products (such as interleukin (IL)-1β and tumor necrosis factor-α (TNFα)) into naive animals, can induce symptoms of neuropathic pain.^17, 18, 19^

The downstream effects of enhanced glial reactivity are strengthened by the fact that immune mediators, including those released by glia, are substantially more potent in modulating neuronal signaling compared with classical neurotransmitters on a per molecule basis.^11^ Glial proliferation, morphological changes and increases in protein expression can persist for months after initial injury, even beyond tissue healing.^20, 21^ Moreover, proinflammatory mediators and glial-derived neurotransmitters can reciprocally stimulate glia in an autocrine and paracrine manner, thereby amplifying a positive feedback loop of unfavorable activity.^22, 23, 24^

### How do glia become activated?

Glia function as a product of their microenvironment, and as such the types of receptors they express vary from site to site, and many receptors can be upregulated to make glia more ‘tuned' to ongoing stimulation. Within the spinal cord, microglia are sensitive to ATP that binds to ionotropic (for example, P2X4 and P2X7) and metabotropic (for example, P2Y6 and P2Y12) purinergic receptors.^25, 26, 27, 28^ Chemokine receptors, such as CX3CR1 (with CX3CL1/fractalkine as ligand) and CCR2 (activated by CCL2/MCP-1), also contribute to the microglial proinflammatory response,^29, 30, 31, 32^ as well as receptors for the sensory neuropeptide, calcitonin gene-related peptide (CGRP)^33^ and interferons (IFN), such as IFNγ.^34^ Akin to microglia, astrocytes can respond to ATP via the surface expression of P2X7 (refs 35, 36) and P2Y1 (refs 25, 37) and can be stimulated by IFNγ,^38^ CGRP^39, 40, 41^ and several mediators released by microglia themselves, including TNFα and IL-18 (for reviews, see refs 11, 42). There is also evidence that astrocytes express tachykinergic NK1 receptors,^43^ with substance P potentiating the IL-1β-mediated induction of IL-1β and prostaglandin E2 (PGE2) secretion from spinal cord astrocytes.^44^

Furthermore, a receptor family expressed by both glial cell types that has gained much recent attention, with regard to pain and immunity, are the Toll-like receptors (TLRs).^45^ TLRs allow glia to sense the presence of pathogen- or microbial-associated molecular products. Importantly, some receptor subtypes, such as TLR4, can additionally recognize endogenous ‘self' warning molecules. Numerous putative ligands have been identified for these so-called damage-associated molecular patterns in the processing of pain, including high mobility group box 1 protein,^46, 47, 48^ heat-shock protein 90 (ref 49) and fibronectin.^50^

### What proinflammatory products do glia release upon activation?

Glial-induced upregulation of proinflammatory signaling is achieved through the induction of gene expression by numerous second messenger-mediated pathways. This includes activation of transcription by phosphorylation of mitogen-activated protein kinases and nuclear factor-κB. Specifically, the mitogen-activated protein kinases implicated here are p38 in microglia,^51^ c-Jun N-terminal kinase in astrocytes^52^ and extracellular signal-regulated kinases (ERKs) in both glial cell types.^53, 54^ The proinflammatory products subsequently released from microglia include IL-1β, IL-6, IL-18, TNFα, PGE2, nitric oxide and brain-derived neurotrophic factor, and IL-1β, IL-6, TNFα, IFNγ, CCL2, CXCL1, CXCL21 and MMP9 from astrocytes (for reviews, see refs 55, 56, 57, 58). In addition, astrocytes can increase their release of gliotransmitters, such as ATP,^59^ glutamate and d-serine.^60^

As the discovery of neuroimmune contributions to pain more than two decades ago,^61, 62, 63^ knowledge of glial-mediated molecular alterations in central sensitization has grown exponentially. Overall, their proinflammatory effects enhance excitatory tone and synaptic efficiency, thereby facilitating an exaggerated pain state. The sequelae of mediators released and resultant outcome are now realized to be highly dependent on the type of glial cell that is activated, the degree of its reactivity and the nature of the stimulus.^64, 65^ For this reason, we will provide a brief summary of the major known excitatory and inhibitory adaptations, and strongly encourage readers to explore other excellent in-depth reviews.^11, 14, 15, 42, 66, 67^

## Glia enhance excitatory nociceptive signaling

**Figure 1:**
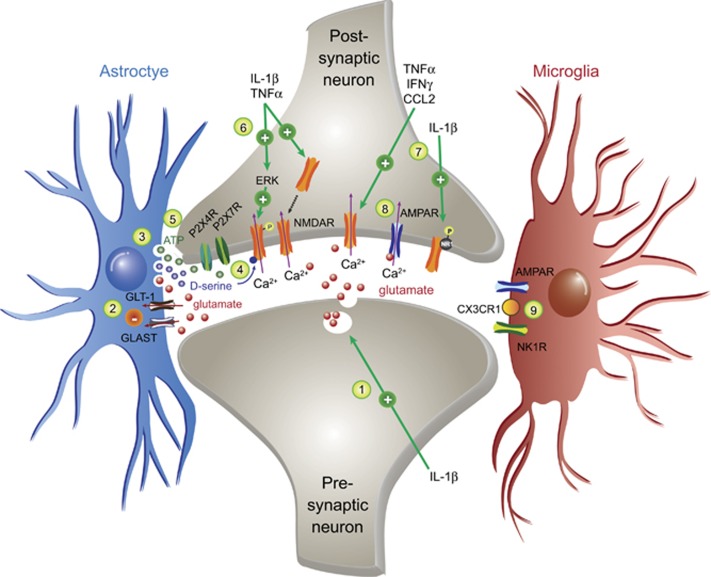
Schematic representation of the major proinflammatory glial-mediated alterations to excitatory synapses within the spinal dorsal horn that contribute to central sensitization. Strong or long-term noxious activation of astrocytes and microglia within the spinal dorsal horn can lead to the aberrant synthesis and release of proinflammatory mediators, such as TNFα and IL-1β. The overarching effect of these neurokine signals in excitatory synapses contributes to central sensitization and facilitates the transmission of nociceptive signals to the brain. Some of the major known adaptations include the following. (1) Increased release of the excitatory neurotransmitter, glutamate, from presynaptic nerve terminals. (2) Suppression of astrocytic glutamate reuptake via downregulation of GLT-1 and GLAST activity. (3) Release of the glutamate from astrocytes, which is capable of increasing the excitability of nearby neurons. (4) d-serine, also released from astrocytes, enhances Ca^2+^ influx via binding to glycine sites on NMDA receptors on postsynaptic neurons. (5) Astrocytic release of ATP also increases postsynaptic excitability via activation of ligand-gated purinergic receptors, P2X4R and P2X7R. (6) TNFα and IL-1β increase translocation of NMDA receptors to the postsynaptic membrane and increases their conductance via an ERK-dependent pathway. (7) IL-1β, TNFα, IFNγ and CCL2 increase NMDA receptor-mediated excitatory signaling; in the case of IL-1β, this is thought to involve the phosphorylation of receptor subunits including NR1, 2a and 2b. (8) Proinflammatory cytokines have been linked to increased expression and activation of AMPA receptors at excitatory synapses. (9) Reactive microglia have increased expression of receptors for various neurotransmitters and chemokines (for example, AMPARs, NK1Rs and CX3CR1), which can induce the further release of proinflammatory cytokines upon stimulation, thereby perpetuating neuronal excitation. ERK, extracellular signal-regulated kinase; IFN, interferon; IL, interleukin; TNFα, tumor necrosis factor-α.

Glial-derived proinflammatory mediators enhance nociceptive signaling in the spinal cord first by facilitating glutamatergic neurotransmission (Figure 1). IL-1β has been shown to increase presynaptic release of glutamate,^68^ and IL-1β, TNFα, CCL2 and IFNγ increase postsynaptic N-methyl-D-aspartic (NMDA) and AMPA receptor currents.^69, 70, 71, 72, 73, 74^ Postsynaptic neurons may further be excited by the release of glutamate from reactive astrocytes.^75, 76^ TNFα can increase postsynaptic NMDA and AMPA-mediated activity by trafficking more receptor to the cell surface,^77^ and by increasing subsequent Ca^2+^ conductance through phosphorylation of neuronal ERK.^78^ In addition, IL-1β can induce SRC-1-mediated phosphorylation of the NR1 subunit on NMDA.^79, 80^ d-serine, a powerful neuromodulator released by reactive astrocytes, enhances depolarizing NMDA cation currents by binding to the NMDAR glycine site.^81^ There is also a persistent decrease in astrocytic expression of GLAST and GLT-1;^82, 83^ loss of function of these glutamate transporters causes an elevation in extracellular glutamate concentrations within the synapse.^84, 85^ Thus, the resultant aberrant uptake and/or release of glutamate, as well as the enhanced activity of its postsynaptic receptors, can contribute to excessive nociceptive signaling reaching the brain.

In addition, increased exocytosis of ATP from reactive astrocytes^42^ can directly stimulate neuronal excitation^86^ or induce glutamate release from presynaptic neurons,^87^ an effect that is facilitated by the upregulation of purinoceptors, such as P2X4R,^50, 88^ P2X7R^89, 90^ and P2Y12R.^91, 92^ Levels of other cytokine and chemokine receptors are also upregulated, including IL-6-induced microglial CX3CR1 (refs 29, 93) that enhances pain via IL-1β.^94^ Under certain conditions, such as IL-1β stimulation, both glial cell types may increase NK1-receptor expression.^95^ This potentiates the response to substance P,^43^ in turn facilitating the release of astrocytic ATP^59^ and proinflammatory cytokines, including TNFα, IL-6 and PGE2.^44, 96, 97^ Last, TNFα, IL-1β and IL-6 can elicit long-term synaptic plasticity by inducing the phosphorylation of the transcription factor cAMP response element-binding protein (CREB),^70^ which may lead to the CREB-mediated transcription of COX-2 and NK1.^98, 99, 100^

## Glia attenuate the inhibition of nociceptive signaling

**Figure 2:**
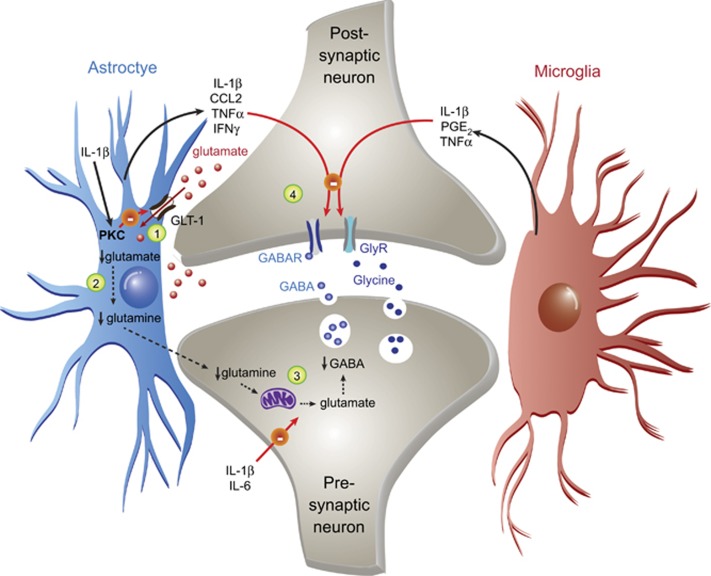
Schematic depicting the major proinflammatory glial-mediated changes to inhibitory synapses within the spinal dorsal horn that facilitate central sensitization. As mentioned in Figure 1, prolonged stimulation of astrocytes and microglia can lead to the increased synthesis and release of various proinflammatory cytokines and chemokines. Within inhibitory synapses of the spinal cord dorsal horn, the effects of these mediators ultimately lead to a reduction in inhibitory neurotransmission (‘disinhibition'), which further facilitates central sensitization. For example: (1) IL-1β can mediate a decrease in the astrocytic uptake of glutamate, via a PKC-mediated suppression of glutamate transporter GLT-1. (2) The reduced uptake of glutamate via GLT-1 leads to decreased availability of glutamine for GABA synthesis. (3) IL-1β and IL-6 inhibit presynaptic GABA and glycine currents. (4) Last, IL-1β, PGE2, CCL2, TNFα and IFNγ decrease GABA and glycine receptor activity; in the case of IL-1β, this is thought to be mediated via a PKC-dependent pathway. IFN, interferon; IL, interleukin; PKC, protein kinase C; TNFα, tumor necrosis factor-α.

Heightened glial activation can also induce disinhibition; that is, a loss of inhibitory signals within the CNS that usually suppress nociceptive transmission, such as GABA and glycine signaling (Figure 2). The activation of microglial TLR4 by lipopolysaccharide (LPS) in rodent spinal slices induces IL-1β release, which suppresses postsynaptic GABA receptor function through the activation of protein kinase C.^101^ IL-1β-induced protein kinase C activation also attenuates astrocytic GLT-1 activity, leading to increased glutamate within the synaptic cleft.^101^ This not only drives a sustained excitation of postsynaptic neurons, but also a deficiency in the supply of glutamine, which is metabolized from glutamate following its reuptake. Consequently, glutamate–glutamine cycle-dependent GABA synthesis by the presynaptic neuron is attenuated.^102^ Moreover, TNFα can prevent action potentials in inhibitory presynaptic neurons;^103^ IL-1β and IL-6 suppress presynaptic GABA and glycine currents;^70^ and PGE2, CCL2 and IFNγ can attenuate postsynaptic electrical activity mediated by GABA or glycine.^104, 105, 106^ Thus, suppression of inhibitory influences within the spinal cord by glial-derived factors may exacerbate pain, by potentiating the transduction of nociceptive information.

## Female sex hormones and neuronal hypotheses underlying the sexual dimorphism of pain

In addition to many pain syndromes having greater prevalence in females than males, other anecdotal evidence suggests that sex steroid hormones can have a direct influence on somatic and visceral persistent pain. In women, for instance, certain painful conditions typically occur during the menstrual years, and symptoms tend to fluctuate with the menstrual cycle.^107, 108^ Symptom severity of several visceral pain conditions, such as irritable bowel syndrome, has been reported to decrease following menopause,^109^ and increase with hormone replacement therapy in postmenopausal women.^110^ Similarly, nociceptive stimuli in rodent visceral pain models are sensitive to both the changing steroid hormone levels throughout the estrous cycle,^111, 112, 113^ and during hormone supplementation following ovariectomy.^114, 115, 116^ Thus, it has been suggested that either elevated or fluctuating levels of sex hormones have a key role in exacerbating persistent pain.^117^

However, the mechanisms underlying this modulation remain unclear and, to date, much of the research has focused on sex steroid-mediated alterations in neural activity and/or molecular targets expressed by neurons. For example, antagonism of neuronal NMDA receptors, often co-expressed with estrogen receptor α (ERα), can attenuate the visceromotor reflex to colorectal distension with greater potency in untreated ovariectomized rats, compared with those with estradiol replacement.^118^ Colorectal distension is correlated with an increase in PKA-mediated NMDAR NR1 subunit expression and phosphorylation in ovariectomized, estrogen-supplemented animals, compared with those not receiving estrogen.^118^ Furthermore, intrathecal administration of estrogen or an ERα-selective agonist can cause an increase in distension-evoked dorsal horn neuron pERK expression, and reverse the decrease in distension-evoked visceromotor reflex produced by ovariectomized rats.^119^

## Does female sex hormone modulation of glial reactivity contribute to the female predominance of persistent pain?

Despite our understanding of the tetrapartite synapse in facilitating nociceptive signaling, it is likely that the contribution of glia has not yet received sufficient attention with regard to the female susceptibility to persistent pain. Intriguingly, TLRs - which, as discussed previously, are one receptor family expressed by glia and have an important role in the immunological response to pathogenic stimuli—are well situated to serve as an important molecular target for persistent pain conditions. This is particularly true for hormonally regulated female pain, as estrogen appears to influence TLR4-mediated proinflammation and pain in various conditions. For instance, glucuronide metabolites (which typically have a longer half-life than the parent molecule) of estrogen cause potent activation of TLR4 *in vitro*, correlating with enhanced mechanical allodynia in rats *in vivo*.^120^ The proinflammatory response to LPS is potentiated by estrogen in female but not male neonatal microglia.^121^ Moreover, although adult hippocampal microglia from ovariectomized rats in *ex vivo* preparations show a downregulation in LPS-induced inflammation upon estrogen supplementation, IL-1β mRNA is potentiated when estrogen is administered *in vivo*.^121^ Long-term estrogen exposure in ovariectomized mice promotes the expression of inflammatory mediators by CNS and peritoneal macrophages, in response to LPS activation *in vivo*^122^ and *ex vivo*,^123^ respectively. Intravenous administration of LPS in humans induces a similar decrease in visceral and musculoskeletal pain thresholds, although intriguingly a much more pronounced increase in circulating levels of plasma TNFα and IL-6 was evidenced in females compared with males.^124^ A recent randomized control trial additionally showed that low-dose LPS was perceived to increase pain from supra-threshold noxious thermal stimuli in women only, and impaired conditioned pain modulation, a measure of endogenous pain inhibition.^125^

Other studies have reported that TLR-mediated responses are important in male but not female pain. Using LPS-induced (in TLR4 mutant mice)^126^ and spinal nerve ligation (in TLR4 knockout mice)^127^ models of pain enhancement, it was reported that mechanical allodynia is TLR4-dependent in males but TLR4-independent in females. Inhibition of spinal p38 MAP kinase has been effective in attenuating inflammatory and neuropathic pain in male, but not female mice.^128^ It has further been proposed that female pain is independent of microglia in a rodent model of mechanical allodynia, alternatively involving the recruitment of T cells.^129^ However, this argument bears further consideration given that males are comparable to females in the generation of autoimmune T cells, but the phenotype of regulatory T cells (Treg), which serve to suppress inflammatory processes, may be more aggressive in males.^130^

Perhaps these opposing results mirror the highly complex, and well recognized, nature of estrogen being both a pronociceptive and antinociceptive hormone (see reviews in refs 131, 132, 133, 134, 135). Regardless, it is evident that the effects of female sex hormones on TLR4-mediated signaling are multifaceted and, given the range of receptors and pathways utilized by glia, highlight the need for research into neuroimmune mechanisms that may be specific to pain in females.

## Somatic versus visceral pain

Persistent pain is a cardinal feature of chronic inflammation of peripheral tissues; thus, our increase in knowledge of neuroimmune signaling has led to investigations of the link between glia and persistent pain associated with inflammation. These data have been primarily acquired using animal models of neuropathic and somatic inflammatory pain, with considerably less attention given to pain arising from the viscera. Although there are many commonalities in the processing of somatic and visceral pain, there are also several important clinical distinctions (for reviews, see refs 136, 137, 138). For instance, pain cannot be evoked from all viscera; visceral pain is diffuse and poorly localized, owing to relatively few visceral afferents with extensive receptive fields; visceral pain can often be referred to remote locations, attributable to visceral and somatic afferent pathways converging into shared spinal levels; injury to the viscera does not necessarily cause pain; and intense motor and autonomic reflexes, such as nausea and muscle tension, usually accompany visceral pain. This aside, the fundamental mechanisms leading to the perception of somatic and visceral pain are similar, where enhanced activity from peripheral nociceptors activates ascending central pathways to the brain. Consequently, the involvement of neuroimmune signaling in persistent pain attributed to visceral inflammation has gained interest in the past few years.^139^

## Neuroimmune contributions to the female predominance of pain associated with inflammation of the pelvic viscera

The viscera are also where sex divergences in pain processing become particularly intriguing, owing to the unique organization of the reproductive and pelvic anatomy in males and females. It has been estimated that women are at greater risk of developing persistent pain within the pelvis, currently affecting between 15 and 24% of women^140, 141^ (versus 1.8–12% in men^142, 143^), including pain due to menstruation, intercourse, pregnancy and childbirth, and infection and inflammation via the vagina, cervix and uterus.^3, 144, 145^ Spinal microglia been found to contribute to pain in male animals with chronic prostatitis.^146, 147^ To our knowledge, however, there are currently no comprehensive studies investigating glial contributions to pain associated with visceral diseases that have been restricted to, or with a substantial focus on, females. This alternative scope in research could reveal distinct female pain mechanisms that may be exploited to improve pain management.

Potential neuroimmune contributions to three visceral conditions that have a greater prevalence in, or are exclusive to, females are discussed below: inflammatory bowel disease (IBD), painful bladder syndrome and endometriosis. These pathologies share several features of neuropathic pain and somatic inflammation, such as heightened neural activity, decreased pain thresholds and increased pain behavior, indicating that central neuroimmune adaptations are probably taking place. This is supported by evidence demonstrating that experimentally induced IBD, cystitis or endometriosis can result in the sensitization of adjacent pelvic organs (for example, intestines, bladder and uterus).^148, 149, 150, 151^ A similar phenomenon is observed clinically with the clustering of comorbidities in women with pelvic pain, such as patients with irritable bowel often presenting with viscero-visceral (for example, bladder or menstrual pain) or viscero-somatic (for example, pelvic muscle spasm, temporomandibular pain) complaints.

### Inflammatory bowel disease

IBD comprises ulcerative colitis and Crohn's disease, both of which involve colonic inflammation; however, each has distinctive pathologic features.^152^ Although the prevalence of ulcerative colitis in males and females is generally similar, the female–male ratio of Crohn's disease in adults is increased up to approximately 1.2–1.3 times.^153, 154^ The studies on glia and IBD have utilized rodent models of di- or trinitrobenzene sulfonic acid-induced colitis, and potential differences between the sexes have not been analyzed.^155, 156, 157, 158^ Nonetheless, marked increases in reactivity were described for microglia in the spinal cord and hippocampus,^155, 156^ and activated satellite glia in the dorsal root ganglia.^156^ This is associated with an upregulation of TNFα levels,^155, 156^ and closer apposition between satellite glial cells and primary afferent neurons in the dorsal root ganglia^156^ via enhanced neuron–glia gap junction coupling.^158^ Associated centrally derived hyperalgesia was assessed by various methods, including increased visceromotor reflex activity^156^ and abdominal withdrawal reflex,^157^ to graded colonic distension. Intracerebroventricular,^155^ intrathecal or systemic^156^ minocycline or intrathecal administration of an anti-TNFα antibody^157^ attenuated the respective pain behaviors examined.

### Painful bladder syndrome

Contributions of neuroimmune overactivity to persistent pain have also been suggested in animal models of, and human patients with, painful bladder syndrome. Formally known as interstitial cystitis, painful bladder syndrome affects approximately 3–7% of adult females and 2–4% of males, encompassing a range of bladder disorders that involve persistent pelvic pain or discomfort, nonspecific urinary symptoms and often cystitis.^142, 159, 160^ In a preliminary study using pooled data from male and female cats with spontaneous feline interstitial cystitis, the fluorescent intensity and number of GFAP-immunopositive astrocytes in the S1 spinal cord dorsal horn was increased compared with healthy unaffected cats.^161^ In addition, it has recently been demonstrated that peripheral blood mononuclear cells from women with painful bladder have an increased proinflammatory response to TLR2 and TLR4 stimulation *in vitro*.^162^ The magnitude of the proinflammatory response also positively correlated with the extent of pelvic and extra-pelvic pain, and the manifestation of comorbid conditions.^163^ This observation has great importance, as the TLR responsivity of peripheral blood mononuclear cells could serve as a neuroimmune biomarker for persistent pain,^164^ given the functional similarities between TLR signaling of immune cells in the periphery and in the CNS. Thus, the heightened TLR responsivity of peripheral immune cells in females with painful bladder syndrome may indicate that CNS sensitization involving neuroimmune modulation may be occurring in parallel, and remains to be explored further.

### Endometriosis

Endometriosis is an estrogen-dependent, chronic, inflammatory medical condition in women, defined as the presence of endometrial tissue in extra-uterine locations, and commonly associated with painful pelvic symptoms. It affects an estimated 5–10% women of reproductive age,^165^ and up to 60% women with persistent pelvic pain.^166^ Endometriosis-associated pain is thought to solely arise from the presence of lesions, yet pain symptoms attributed to the disease can occur in women with lesions removed,^167^ and the severity of experienced pain correlates poorly with the degree of lesions.^168, 169^ Thus, it exemplifies all that is female, from the unique visceral anatomy to the complex hormonal interplay, and the long-standing association with unexplained persistent pain.

Given that the conditions mentioned above affect the visceral organs present in both sexes, studying endometriosis (and indeed other female-specific conditions, such as vulvodynia) may provide further insight into subpopulation adaptations of neuroimmune-mediated pain. Neural changes have been studied in detail,^170, 171^ and it has been suggested that pain attributed to endometriosis is likely to involve neuronal processes leading to central sensitization.^115, 170, 172, 173^ However, a potential role for glia has yet to be investigated. Accumulating evidence nevertheless demonstrates that there are alterations in peripheral immune function in endometriosis patients.^174, 175^ LPS-stimulated peritoneal macrophages from women with endometriosis secrete significantly higher levels of proinflammatory cytokines (for example, IL-6 and TNFα) than non-diseased counterparts, an effect that can be attenuated by pre-treatment with a TLR4-neutralizing antibody.^176^ TLR4 mRNA transcript expression is increased up to sixfold in endometriosis lesions compared with eutopic endometrium,^177^ and TLR2 and TLR9 mRNA from peritoneal effusions are upregulated in endometriosis patients compared with healthy controls.^178^ It remains to be determined whether the increased TLR levels are owing to an upregulation of the receptors per immune cell, or recruitment of TLR-bearing cells to the diseased area. There is now also solid evidence from multiple lines of investigation that the development and maintenance of endometriosis involves atypical peritoneal macrophage activity.^179, 180^

Collectively, these data suggest that several alterations in neural, immune and neuroimmune functions exist in the female-predominant conditions of IBS, painful bladder and endometriosis. Studies that further investigate visceral disease-associated modifications in neuroimmune signaling are desirable. Such information would further our knowledge of persistent pain mechanisms, and may also identify a molecular basis of pain susceptibility in the subpopulation of females.

## Does the dorsal root reflex and neurogenic inflammation contribute to the development of visceral inflammatory conditions?

Besides painful symptoms, many chronic inflammatory diseases present with visible tissue abnormalities and consequently a vast number of studies focus on characterizing and treating these lesions. However, attention has recently shifted to unraveling the complex molecular pathways that instead underlie disease etiology. This is particularly interesting in the example of endometriosis, which is generally attributed to the movement of menstrual debris through the fallopian tubes into the abdominopelvic cavity during menses (retrograde menstruation).^181^ Although it is estimated that approximately 90% women aged 15–49 years will exhibit retrograde menstruation,^182^ only around one in ten will develop endometriosis lesions. Similarly, in many patients, the onset of IBD follows a bout of gastroenteritis,^183^ yet not all individuals with gastroenteritis will develop IBD. Thus it seems other factors affect the likelihood of disease formation in subsets of patients, leaving them susceptible to developing disease compared with their peers.

It is well established that sensitized sensory nerves can initiate or exacerbate inflammatory conditions by the release of neuropeptides from peripheral nerve terminals, such as CGRP and substance P.^184, 185, 186^ This results in edema, immune cell infiltrate and other sequelae reminiscent of inflammation; hence has been termed neurogenic inflammation.^187^ The release of such peptides in the periphery is known to occur via two antidromic signaling mechanisms. Initially, there is strong local stimulation of peripheral nerve terminals at the site of disease, known as the ‘axonal reflex'. With increased afferent input, the central terminals of sensory neurons within the spinal dorsal horn may also be excited, leading to anterograde propagation of action potentials back to the periphery (the ‘dorsal root reflex').^188, 189, 190^

Centrally derived neurogenic inflammation via the dorsal root reflex contributes to pathology in several animal models of peripheral inflammation, mostly involving the skin^191, 192, 193, 194, 195, 196^ and joints,^197, 198, 199^ but also colitis.^200^ Compared with control animals receiving infused saline, colonic tissues from rats stimulated with intrathecal SP to the lumbar spine showed increased protein expression of the proinflammatory cytokine, migration inhibitory factor, mucosal edema and lymphocyte infiltration, effects that were attenuated by intrathecal pre-treatment with an NK1-receptor antagonist. The efferent propagation of inflammation via central dorsal horn activation has also been supported in humans, by observations that relapses in ulcerative colitis have been associated with electrical stimulation of the spinal cord.^201, 202, 203^

## Does central glial stimulation and overactivity trigger peripheral neurogenic inflammation of the viscera?

In addition to neuropeptides, it has been suggested that proinflammatory cytokines are able to stimulate dorsal horn afferents to influence the development of peripheral inflammation.^204, 205^ It has been reported that spinal IL-1β, associated with reactive astrocytes, can contribute to the induction and maintenance of temporomandibular arthritis and associated pain.^205^ In these experiments, central disruption or inhibition of spinal IL-1 receptor type 1 (a receptor for IL-1β) signaling in mice with established arthritis, resulted in significant attenuation of joint pathology. Mice without previously established arthritis showed an upregulation of astrocyte reactivity within the dorsal horn following local spinal overexpression of IL-1β, as well as joint changes indicative of the initial stages of arthritic disease. Enhanced CGRP expression was observed in primary sensory fibers of mice with IL-1β-overexpression (peripheral projections, dorsal root ganglia and central projections), which also displayed spontaneous behavior indicative of pain. It was suggested that bidirectional crosstalk between the CNS and peripheral joints, via spinal IL-1β stimulation of sensory afferents to release CGRP in the periphery, may have a role in the exacerbation of inflammation and pain.^205^ Therefore, heightened spinal glial reactivity and proinflammatory signaling may contribute to ongoing peripheral inflammation, as well as enhancing pain by central sensitization.

**Figure 3:**
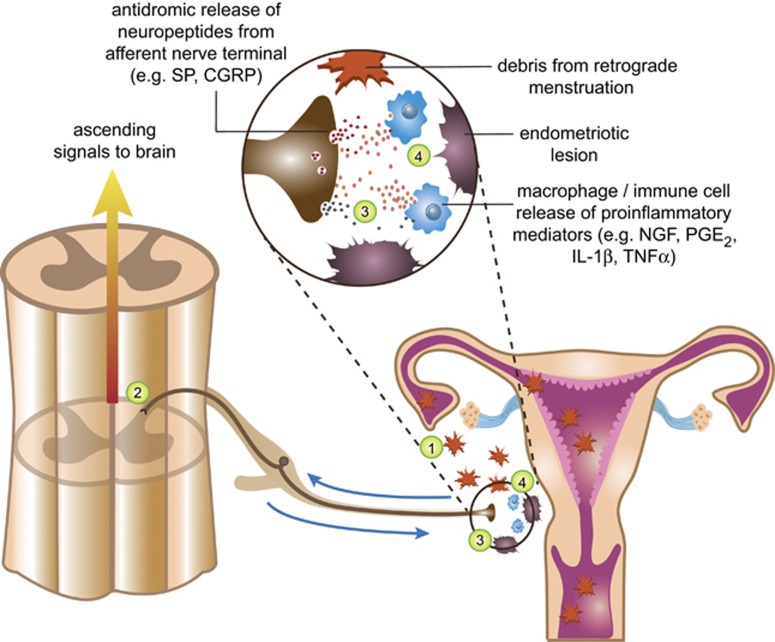
Possible involvement of centrally mediated neurogenic inflammation in the development of visceral inflammatory disease in the periphery: example for endometriosis. (1) During menstruation, endometrial debris passes both per vaginum and in a retrograde fashion through the fallopian tubes to the peritoneal cavity. (2) In certain women, the inflammatory events initiated by ectopic endometrial tissue activate sensory afferents innervating adjacent visceral structures, which transmit the noxious information to the spinal dorsal horn. In addition to exciting ascending neural signals projecting to the brain, afferent neurotransmitter release could potentially also activate spinal astrocytes and microglia, whose proinflammatory products contribute to the development of central sensitization and exaggerated pain (see Figures 1 and 2 for details). (3) Strong ongoing afferent stimulation associated with regular monthly menstruation and dysmenorrhea, as well as the excitatory environment created by reactive glia, may reciprocally activate the central terminals of sensory nerves. This can then induce the antidromic release of neuropeptides (such as SP and CGRP) at the peripheral site of disease (the ‘dorsal root reflex'). (4) The subsequent induction of neurogenic inflammation, including the release of cytokines (IL-1β and TNFα), PGE2 and nerve growth factor (NGF) from local immune cells, may then contribute to an environment that encourages the implantation of endometrial debris onto the peritoneum, and the development of endometriotic lesions (including the associated neovascularization and sprouted innervation). CGRP, calcitonin gene-related peptide; IL, interleukin; PGE2, prostaglandin E2; TNFα, tumor necrosis factor-α.

This raises the interesting question as to whether centrally derived neurogenic inflammation, generated in part by neuroimmune signaling, contributes to the perpetuation of other inflammatory diseases. Indeed, neurogenic inflammatory processes have been implicated in the exacerbation of IBD, cystitis and endometriosis.^206, 207, 208, 209^ In endometriosis, neurogenic inflammation is thought to create an optimal peritoneal environment for ectopic lesion formation in the visceral tissues.^210, 211^ In this setting, enhanced afferent signaling in response to accumulating endometrial debris may facilitate lesion development by a positive feedback loop (Figure 3). Further research into the role of glia and the dorsal root reflex in the development of inflammation are recommended.

## Early-life stressors as central glial primers for visceral inflammation

It is now realized that glia have the ability to be ‘primed' by prior experience to over-respond to new immune challenges (a ‘two-hit hypothesis'^14^). This is shown where laparotomy and intraperitoneal injection of LPS each individually cause modest increases in mechanical allodynia. However, allodynia is potentiated up to threefold when laparotomy and LPS are administered sequentially, with enhanced pain being associated with heightened microglial reactivity.^212^

Many studies are currently investigating the impact of early-life stressors, such as maternal separation or injury, on long-lasting glial alterations in the adult. Such events can be the ‘first hit' that primes glia to over-respond and be detrimental in restoring ‘second hit' immune challenges later in life. Visceral hyperalgesia can be enhanced by early adverse events,^213, 214, 215, 216^ although associations with glia have thus far been described only for somatic pain. For instance, incisional surgery of the neonatal rat hind paw caused an increase in the intensity of microglial activation and expression within the dorsal horn that persisted into adulthood.^20^ This was associated with hyperalgesia following incisional surgery as an adult, and was prevented by intrathecal administration of minocycline at the time of adult injury. Thus, this suggests that early adverse life events provoking long-term heightened glial reactivity may lead to greater sensitivity to future harmful stimuli.

Priming of spinal glia may provide an explanation for why some subpopulations, such as females, are predisposed to developing certain painful conditions. If the neuroimmune communication has been primed before a persistent pain-triggering insult, then this mechanism may inherently increase disease burden in females (or males) due to the increased release of proinflammatory products, and may also be exacerbated by the activity of sex hormones, such as estradiol. Early aggravation of spinal glia might therefore contribute to the development of peripheral inflammation, via the dorsal root reflex or otherwise. Regarding endometriosis, clinical records from female monkeys have indicated that animals exposed to prior adverse life events, such as laparoscopic examination and cesarean section, were associated with an increase in the incidence of developing endometriosis.^217, 218^ The initial scenario of gastroenteritis preceding IBD could further represent the ‘first hit' of irritation that sensitizes the neuroimmune system, later contributing to disease progression. Direct evidence linking early-life glial priming and the incidence of visceral inflammation in adulthood await to be studied.

## Beyond ‘hysteria' towards targeted treatment of female pain

Our current understanding of central sensitization leading to the development of persistent pain involves interactions between neurons and highly reactive glia. Studying alterations in these neuroimmune connections under various conditions provides enormous potential for meaningful new research discoveries and, given the significant female predominance of pain, may contribute to understanding the biological mechanisms that underlie sex differences in pain processes. Using both male and female subjects will be crucial for this future pain research. Exploring painful conditions of the viscera that are most prevalent or specific to each of the sexes, such as IBD, painful bladder syndrome and endometriosis in females and prostatitis in males, may additionally provide clues into the unique anatomical and neuroendocrine influences on pain sensitivity. Indeed, the potential contribution of neuroimmune and neurogenic signaling to inflammation and pain is a novel avenue for gynecological and urogenital research. Although much of this review has focused on female sex hormones and pain, male sex hormones may also have a critical role, where low testosterone levels are an emerging link to persistent pain states in both the sexes.^219, 220^ Thus, prospective studies comparing the root causes of sex-specific pain conditions may have important implications for both future pain prevention and treatment strategies.

As we unravel the molecular pathways involved in enhancing nociceptive transmission, this will provide opportunities for resultant drug discovery. New pharmacotherapies that aim to target glia to modulate their deleterious, proinflammatory contributions to pain are now steadily emerging.^14, 221^ This is emphasized by recent exciting studies that have for the first time demonstrated an upregulation of central glial cell reactivity in pain patients *in vivo*.^222, 223, 224^ Although the translation of results from animals to humans has been variable in effectiveness, an issue plaguing the field of pain at large,^225, 226^ it is likely that the future analgesic success of these agents will be highly dependent on the type of injury or disease, the selection of drug and dosing regimen, the route of delivery and the timing of treatment. With continued investigations, the neuroimmune system represents a key target to decrease the burden of persistent pain.
